# Referrals for positive tuberculin tests in new health care workers and students: a retrospective cohort study

**DOI:** 10.1186/1471-2458-10-28

**Published:** 2010-01-20

**Authors:** Yining Xu, Kevin Schwartzman

**Affiliations:** 1 Montreal Chest Institute, Rm K1.21, 3650 St. Urbain St, Montreal, Quebec, Canada H2X 2P4; 2 McGill University, Montreal, Quebec, Canada

## Abstract

**Background:**

Documentation of test results for latent tuberculosis (TB) infection is important for health care workers and students before they begin work. A negative result provides a baseline for comparison with future tests. A positive result affords a potential opportunity for treatment of latent infection when appropriate. We sought to evaluate the yield of the referral process for positive baseline tuberculin tests, among persons beginning health care work or studies.

**Methods:**

Retrospective cohort study. We reviewed the charts of all new health care students and workers referred to the Montreal Chest Institute in 2006 for positive baseline tuberculin skin tests (≥10 mm). Health care workers and students evaluated for reasons other than positive baseline test results were excluded.

**Results:**

630 health care students and workers were evaluated. 546 (87%) were foreign-born, and 443 (70%) reported previous Bacille Calmette-Guérin (BCG) vaccination. 420 (67%) were discharged after their first evaluation without further treatment. 210 (33%) were recommended treatment for latent TB infection, of whom 165 (79%) began it; of these, 115 (70%) completed adequate treatment with isoniazid or rifampin. Treatment discontinuation or interruption occurred in a third of treated subjects, and most often reflected loss to follow-up, or abdominal discomfort. No worker or student had active TB.

**Conclusions:**

Only a small proportion of health care workers and students with positive baseline tuberculin tests were eligible for, and completed treatment for latent TB infection. We discuss recommendations for improving the referral process, so as to better target workers and students who require specialist evaluation and treatment for latent TB infection. Treatment adherence also needs improvement.

## Background

Tuberculosis (TB) remains a leading cause of morbidity and mortality worldwide. In 2007, there were an estimated 9.3 million new cases of TB and 1.75 million resulting deaths [1,2]. In both developing and developed countries, health care workers and students (HCWS) represent an important risk group for exposure, infection, and potentially, disease [3,4]. Although TB in Canada is rare overall (4.7 cases/100,000 population in 2007) [5], previous studies have highlighted the risks of TB infection faced by Canadian health care workers [6,7]. After a few highly publicized active TB cases in health care workers [8,9], some have called for mass treatment of latent TB infection in this group [9,10].

Current Canadian guidelines for TB screening among HCWS call for a baseline two-step tuberculin skin test (TST). A result of ≥10 mm on the first or second test is considered positive, triggering referral for chest radiography and medical evaluation--to identify persons with active disease, and potential candidates for treatment of latent TB infection (LTBI). All HCWS with negative baseline tests will subsequently undergo repeat skin testing for detection of newly acquired infection, which is a priority for treatment. Repeat skin testing may be done routinely, or after specific TB exposures of concern [10,11]; annual chest radiography is not recommended. Baseline screening is also useful to identify HCWS with positive tuberculin skin tests but little risk for progression to active TB, who can be discharged from further evaluation when appropriate [11,12].

In Canada, the standard treatment for LTBI is 9 months of daily isoniazid (INH), with 6 months of daily INH or 4 months of daily rifampin considered acceptable alternatives [11-13]. These regimens are associated with frequent minor side effects--and rarely, with severe hepatotoxicity, a risk that increases with age[14]. Hence, Canadian HCWS aged ≥35 years with positive baseline tuberculin tests but normal chest radiographs, are not candidates for treatment of LTBI in the absence of other medical risk factors for TB reactivation. More generally, the long treatment course and the frequency of side effects pose substantial challenges in ensuring adherence and successful completion.

In Canada, a further complicating factor is that many HCWS have emigrated from high TB incidence countries: in a multi-centre Canadian survey the overall proportion of foreign-born health care workers was 21%; it was much higher in some cities [15,7]. Foreign-born HCWS may already have acquired latent TB infection even without previous health care work. In addition, interpretation of TST results in the foreign-born may be confounded by previous Bacille Calmette-Guérin (BCG) vaccination, which can cause initial positive TST results, or positive "second-step" (booster) reactions [16,17].

Although previous studies have focused on the yield of screening and treatment of latent TB infection in HCWS [18-24], few studies have specifically addressed the efficiency of baseline TST screening for future HCWS. The aim of this study was to describe the demographic, clinical and radiographic characteristics of HCWS in Montreal with positive baseline tuberculin tests, as well as the diagnostic and treatment outcomes of the referral process.

## Methods

In this retrospective cohort study, we reviewed the standardized report forms for TB referrals at the Montreal Chest Institute (MCI), for positive baseline tuberculin tests among HCWS. We reviewed all such referrals for calendar year 2006, to allow complete capture of all subsequent treatment outcomes. Workers and students evaluated for reasons other than baseline positive tests were excluded. Demographic and clinical data, diagnostic test results, eligibility for treatment of LTBI, and treatment outcomes were entered into a Microsoft Access^® ^database. When the examining physician specifically attributed a positive tuberculin test to BCG vaccination, this was noted. Adequate treatment for LTBI was defined as, at least 6 months of isoniazid or 4 months of rifampin [11], based on prescriptions, clinic attendance, and documentation by physicians and nurses.

We used STATA statistical software (Version SE8, College Station, Texas) for descriptive statistics and bivariate comparisons (Student's t-test for comparison of means, chi-squared test for proportions). Subjects with missing data were excluded from analyses involving the missing variables, but were retained for other analyses. This study was approved by the Research Ethics Board of the McGill University Health Centre.

## Results

A total of 633 persons met study inclusion criteria (Table 1). Over two-thirds were female, and mean age was 33 years. There were 42 subjects (6.7%) who reported previous health care work outside Canada, this included 18 (2.9%) nurses and 11 (1.7%) physicians. Of 124 (20%) who had already begun health care work in Montreal, the professions most frequently listed were paramedic/ambulance technician (37; 5.9%) and orderly (16; 2.5%); current job was not specified for 48 (7.6%). Among the 478 students, the training programs most frequently listed were nursing (87/478; 18%), nursing assistant (58; 12%), orderly (42; 8.8%); there were 12 (2.5%) medical students; and 190 (40%) did not specify program.

**Table 1:** Summary of Patient Characteristics

Patient Characteristics	N (SD or %)
Age, years, mean (SD)	33.4 (9.5)
Sex, Female	437/630 (69%)
Foreign Born	546/630 (87%)
Mean years in Canada, if foreign-born (SD)	8.0 (8.3)
WHO Regions of birth, if foreign born	
Americas	209/546 (38%)
Africa	101/546 (19%)
Eastern Mediterranean	86/546 (16%)
Europe	78/546 (14%)
Western Pacific	63/546 (12%)
South-East Asia	9/546 (2%)
Reason for Screening	
Before health care work	65/630 (10%)*
Before health care studies	478/630 (76%)*
Just started health care work	124/630 (20%)*
BCG vaccination:	
Yes	443/630 (70%)
No	91/630 (14%)
Unknown	96/630 (15%)
Tuberculin Skin Test #1 quantitative result, total	615/630 (98%)
<5 mm^†^	78/615 (13%)
5-9 mm^†^	59/615 (10%)
10-14 mm^†^	211/615(34%)
≥15 mm^†^	267/615 (43%)
Tuberculin Skin Test #2 quantitative result, total	154/630 (26%)
<5^‡^	4/154 (3%)
5-9^‡^	4/154 (3%)
10-14^‡^	81/154 (53%)
≥15^‡^	65/154 (42%)
Any Symptoms	12/630 (2%)
Any Concomitant Medical Conditions	84/630 (13%)
Any Regular Medications	136/630 (22%)
Any Smoking History	85/630 (14%)
Any Physical Examination Finding	18/630 (3%)
CXR: Normal	564/630 (90%)
Minor Abnormality	28/630 (4%)
Inactive TB	28/630 (4%)
Possible Active TB	1/630 (0.2%)
Non TB related Abnormality	6/630 (1%)
Pregnant (No CXR)	3/630 (0.5%)

*Some had more than one reason for screening

Of the 546 foreign-born subjects: 313 (57%) were born in countries with annual TB incidence ≥100 active TB cases/100,000 population, while 184 (34%) were born in countries with an incidence of 25-99/100,000 [1]; 382 (70%) reported prior BCG vaccination; 43 (7%) reported known TB exposure; 9 (1.5%) reported previous treatment for active TB; and 6 (1.0%) reported previous treatment for latent TB infection.

Quantitative results for at least one TST were available for 615 subjects: 478 (78%) had ≥10 mm induration on their first test; and over 90% of the remaining subjects had ≥10 mm induration on a second TST after a first test considered negative. There were 105 (17% of those with quantitative skin test results) who met Canadian criteria for boosting on the second test [11]; and 8 had their first tuberculin test documented simply as "negative," with quantitative results available for the second tuberculin test only.

Nearly all subjects denied any symptoms, but 84 (13%) reported at least one underlying medical condition, most commonly asthma/allergy and hypertension, and 8 (1.3%) reported medical conditions associated with elevated risk of TB reactivation: 6 diabetics, 1 with renal disease, and 1 with HIV infection. Only 28 (4.5%) had radiographs compatible with previous (inactive) TB scarring. One person had a radiograph consistent with possible active TB and underwent further microbiologic investigation, but did not have active disease.

**Figure 1:**
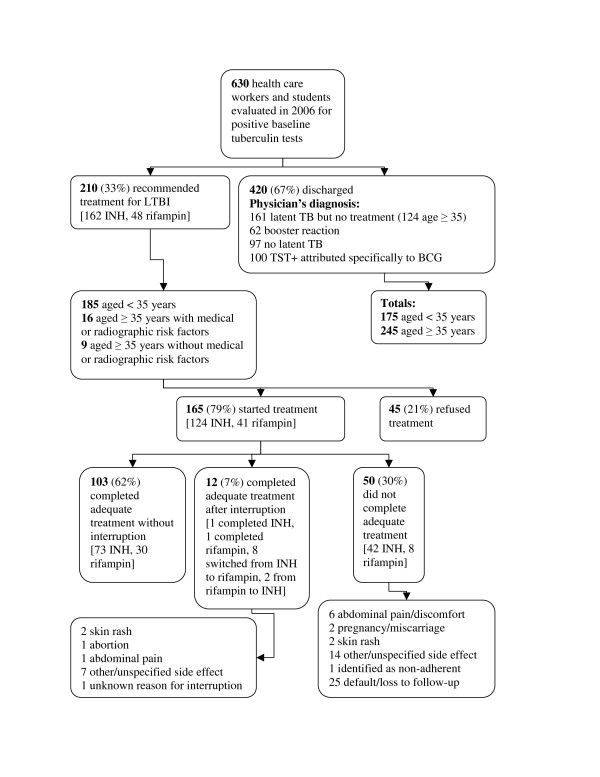
**Health care workers and students evaluated at the Montreal Chest Institute, 2006**. Flow chart of health care workers and students evaluated at the Montreal Chest Institute for positive baseline tuberculin tests, 2006. Abbreviations: LTBI = latent tuberculosis infection, INH = isoniazid, TST = tuberculin skin test, BCG = Bacille Calmette-Guérin.

The physicians' final diagnoses and treatment recommendations are summarized in Figure 1. The 210 patients who were recommended for treatment, were significantly younger than the 161 patients judged to have LTBI but not recommended for treatment (mean 28.0 vs. 39.1 years, P < 0.0001 by Student's t-test). Of the 161 for whom treatment was not recommended, 124 (77%) were aged ≥35 years. Among the untreated group with LTBI: 6 reported previous treatment for active TB, 5 reported previous treatment for LTBI; 1 was about to move away, 1 wished to become pregnant, and 1 had underlying liver disease. There were no other significant demographic features distinguishing treated vs. untreated patients with latent TB. Of note, 9 persons aged ≥35 years were treated despite normal chest radiographs and no relevant medical conditions. These individuals may have requested treatment despite being older than the usual age threshold in this context.

Of those who were offered treatment for latent TB, 21% refused; these individuals were even more likely to report prior BCG vaccination than were those who accepted treatment. Of the subjects who refused treatment, 31 out of 45 (69%) had a history of BCG vaccination, whereas only 83 (50%) of 165 patients who started treatment (P = 0.03 by chi-square test) reported BCG vaccination. Those who refused treatment may have judged that their positive tuberculin tests reflected their prior vaccination, or perhaps they believed that protection by the vaccine made treatment unnecessary [20]. Subjects who refused treatment did not differ from those who began it, with respect to age, sex, foreign birth, TB incidence in country of origin, or duration of residence in Canada (detailed data not shown).

Of 210 HCWS prescribed treatment, 165 (79%) began treatment and 115 (70%) of these completed adequate treatment. Of those prescribed isoniazid, 74 (60%) of 124 completed treatment. Of those prescribed rifampin, 31 (76%) of 41 completed treatment (P = 0.07, chi-square test). Due to side effects, 8 who began isoniazid completed treatment after changing to rifampin, and 2 who began rifampin completed treatment after changing to isoniazid

Treatment interruption or early discontinuation occurred in 62 (38%) of those treated (Figure 1), most often because of default or non-adherence (26 subjects). Subjects who interrupted treatment, or discontinued it early, were of similar age to completers: mean age 27.5 years for interrupters vs. 28.0 years for completers. The proportion of females who interrupted treatment was 38% vs. 23% for males (P = 0.05 by chi-square test). Treatment interruption was no more frequent in subjects aged >35 than in younger subjects (37% vs. 33%). No subject developed clinical hepatotoxicity or transaminases ≥3× the upper limit of normal.

## Discussion

Two thirds of new health care workers and students evaluated for positive tuberculin skin tests were discharged from a tuberculosis referral centre without treatment. The evaluating physicians judged that many tests were false positives reflecting previous BCG vaccination - and that most patients were at low risk of reactivation even with true latent infection. A minority of referrals reflected booster reactions. Radiographic findings which would increase future TB risk were uncommon, and no cases of active TB were identified. One-third of subjects were deemed eligible for treatment of latent infection. Of these, 79% began treatment. Although 70% of subjects who started therapy for latent TB completed their treatment, this was only 18% of all subjects referred.

This was a retrospective study, so we were limited to information documented during routine patient care rather than data collected specifically for our objectives. As we could only review files of those persons who were referred to, and showed up at the MCI, we could not assess the yield of the initial phases of TST reading and referral. We could not estimate the proportion of baseline tests which were positive: the fact that 59% of subjects were considered to have latent TB reflected only HCWS referred for positive tuberculin tests, most of whom came from countries with high TB incidence. We were also unable to compare referred HCWS with peers who had negative baseline tests, and we could not capture individuals who did not make or keep their initial appointments after a positive test.

This study only considered patients seen at a single TB referral centre, and is not representative of all patients or practitioners. It is unlikely that the process is more efficient in primary care clinics or occupational health offices. The rate of treatment initiation was consistent with that reported elsewhere [18]. When subjects with latent TB were not offered treatment, the major reason appears to have been age with attendant toxicity risk, although this was not explicitly documented by the examining physicians. We did account for documented patients' refusal of treatment. However, it remains possible that some patients made it clear from the outset that they would not accept treatment for latent TB; no prescription was offered by the treating physician but the patient's refusal may not have been documented.

A strength of this study was the diverse population of health care workers and students, evaluated consecutively in a real-life practice setting. This differs from clinical trials, where subjects may be unrepresentative with respect to their demographic and clinical profile, and their adherence. Another strength was the physicians' use of a standardized form for patient evaluations. Patients were managed by a small group of physicians and nurses with substantial experience in TB care. Although suboptimal, the 70% completion rate for treatment of latent TB infection compared favourably with previous reported values among health care workers, which have ranged from 38-66% [18,22-24].

In our study, the physicians' approach differed from that advocated by the U.S. Institute of Medicine which recommends that treatment of latent tuberculosis infection should be widely expanded [25]. Salpeter and Salpeter suggested that screening and treatment for LTBI for all HCWS was cost saving, based on optimistic assumptions about cost and completion rates for INH treatment [19]. Based on this study and on earlier cost data from the MCI [26-28] in Montreal, the cost per case prevented through baseline screening and treatment of HCWS was likely to exceed considerably the cost of treating an active TB case.

Nonetheless, beyond the direct cost of treatment there are many good reasons to prevent TB in health care workers--including transmission to other workers as well as patients. However, the yield and efficiency of the referral program would be substantially enhanced by reducing unnecessary evaluation of persons who are not treatment candidates, and by ensuring completion of treatment by those who are.

A more practical and potentially cost-effective approach might be to limit referrals for further evaluation and treatment after a baseline positive tuberculin test, supplemented by a simple clinical questionnaire and chest radiograph. For example, referrals might be limited to 1) the small number of symptomatic individuals; 2) persons with comorbid conditions or radiographic findings that place them at increased risk of future TB disease; 3) persons <35 years of age, who are at lowest risk for treatment toxicity.

One solution would be a protocol for occupational health nurses, whereby asymptomatic workers aged ≥35 with baseline positive tuberculin tests but no medical risks and normal chest radiographs could be discharged - since ordinarily, they would not be treated. This group represented almost 40% of our cohort. Similarly, workers with booster reactions on 2-step tuberculin testing (10% of our cohort) could also be discharged by nurses, if their radiographs are normal and they have no other medical risks. Lambert and colleagues identified personnel costs, training and education as the major cost drivers of a TST screening program [21]. Implementation of a protocol of this type could reduce superfluous evaluations by TB specialists, and reduce costs.

Another alternative is to incorporate interferon-gamma release assays (IGRAs) into the baseline testing algorithm. U.S. guidelines suggest that IGRAs may be substituted for baseline and follow-up tuberculin skin tests among health care workers [11]. These blood tests require only a single testing visit and eliminate confounding by prior BCG vaccination, but are substantially more expensive than the tuberculin skin test [27]. To the extent that they reduce unnecessary testing and treatment, and promote uptake, [29] this could offset the costs of the assays themselves and enhance their cost-effectiveness in this setting [27,30]. Physicians and patients may be more likely to initiate treatment if they trust that BCG effect has been excluded. This is particularly relevant, since in our study prior BCG vaccination was associated with HCWS' refusal to begin therapy.

Revised Canadian guidelines (2008) suggest that tuberculin skin testing should remain the primary screening modality for Canadian workers, with IGRAs reserved for confirmation of positive tuberculin test results in low-risk workers [31]. As with the U.K. NICE guidelines [32], the Canadian guidelines reflect the fact that data are only now emerging, with respect to the positive predictive value of the IGRAs for subsequent development of active TB [33]. Guidelines also reflect the limited information currently available to guide interpretation of serial IGRA results. A recent South African study demonstrated substantial test-retest variability for IGRAs among health care workers, highlighting challenges in interpreting serial tests [34].

For baseline testing, a practical approach would be to confirm TST results ≥10 mm with an IGRA at the time of TST reading [34] for BCG-vaccinated HCWS from lower-incidence countries. Our findings suggest that some "false-positive" referrals could be avoided by using IGRAs to supplement the baseline TST in this manner. Workers with negative IGRA results could then be discharged by the occupational health nurse. More definitive data about the conduct and interpretation of serial interferon-gamma release assays will be particularly important in guiding future practice [35].

## Conclusions

The documentation of tuberculin skin test results is important for all persons beginning health care work. However, in our setting, the yield of current practices for referral and evaluation following baseline positive tests appeared limited. There is room for improvement in targeting evaluation and treatment, and in completion of treatment for latent TB infection when prescribed.

## Competing interests

The authors declare that they have no competing interests.

## Authors' contributions

Yining Xu aided in development of the data collection tools, collected the data, aided in data analysis, and wrote the initial draft of the manuscript. Kevin Schwartzman designed the study, aided in development of the data collection tools, aided and supervised the data analysis, and provided extensive revisions to the manuscript. Both authors have read and approved the final manuscript.

## Pre-publication history

The pre-publication history for this paper can be accessed here:

http://www.biomedcentral.com/1471-2458/10/28/prepub
